# A New Design to Rayleigh Wave EMAT Based on Spatial Pulse Compression

**DOI:** 10.3390/s23083943

**Published:** 2023-04-13

**Authors:** Chuanliu Jiang, Zhichao Li, Zeyang Zhang, Shujuan Wang

**Affiliations:** School of Electrical Engineering and Automation, Harbin Institute of Technology, Harbin 150001, China

**Keywords:** electromagnetic acoustic transducer, Rayleigh waves, spatial pulse compression, unequal spacing coil, wavelength modulation

## Abstract

The main disadvantage of the electromagnetic acoustic transducer (EMAT) is low energy-conversion efficiency and low signal-to-noise ratio (SNR). This problem can be improved by pulse compression technology in the time domain. In this paper, a new coil structure with unequal spacing was proposed for a Rayleigh wave EMAT (RW-EMAT) to replace the conventional meander line coil with equal spacing, which allows the signal to be compressed in the spatial domain. Linear and nonlinear wavelength modulations were analyzed to design the unequal spacing coil. Based on this, the performance of the new coil structure was analyzed by the autocorrelation function. Finite element simulation and experiments proved the feasibility of the spatial pulse compression coil. The experimental results show that the received signal amplitude is increased by 2.3~2.6 times, the signal with a width of 20 μs could be compressed into a δ-like pulse of less than 0.25 μs and the SNR is increased by 7.1–10.1 dB. These indicate that the proposed new RW-EMAT can effectively enhance the strength, time resolution and SNR of the received signal.

## 1. Introduction

Rayleigh waves are widely used for nondestructive testing (NDT) because of their low attenuation and pure wave mode. Because the Rayleigh wave energy is primarily concentrated under the surface of a sample, defects in these areas can be detected efficiently [1]. One of the most common methods to generate and detect Rayleigh waves is by using an electromagnetic acoustic transducer (EMAT) with a permanent magnet and a meander line coil [2]. The EMAT does not require contact with the sample because the ultrasound vibrations are induced directly in the sample via an electromechanical coupling. It is attractive for some situations where using a couplant is difficult, such as high-temperature, high-speed and on-line inspection [3,4,5,6,7].

A variety of methods have been developed to improve the signal-to-noise ratio (SNR) of the Rayleigh wave EMAT (RW-EMAT). Considerable research efforts have been devoted to optimizing the transducer geometry parameters, including both coil and magnet [8,9,10]. These works are a significant benefit for the application of the RW-EMAT for surface defect detection. Ferrite material can also be utilized as a magnetic backplane to increase the intensity of the static magnetic field [11]. Besides the transducer structure, some researchers have optimized the driving circuits for EMATs, such as increasing the pulse intensity [12] and employing matching capacitors to improve the amplitude of resonant voltage [13,14].

The approaches described above have made significant progress to improve the signal intensity and SNR of the RW-EMAT. The coils used in the previous studies are normally meander line coils with equal spacing (ES). Some studies have shown that the meander line coils with unequal spacing (UES) are capable of exciting the focusing waves [15,16,17]. However, the UES coil structure leads to a novel and interesting phenomenon known as spatial pulse compression (SPC).

Pulse compression is a radar-related technique that is used to increase signal intensity and resolution [18,19], which has also been demonstrated in the field of ultrasonic NDT [20,21,22]. Its main aim is to obtain a δ-like signal from a specific excitation waveform via two subprocesses, stretch and compression. Scholars are particularly interested in discovering which form of the excitation signal can provide a final signal with a narrower main lobe width, larger pulse compression ratio and higher SNR [23,24,25]. Some efficient excitation signals, such as the chirp, non-linear modulation and coding excitation signal, have progressively been proposed [26,27,28]. The entire signal conversion process takes place in the time domain, so the technique can be called temporal pulse compression (TPC). These studies about TPC enhance the strength, resolution and SNR of the signal, and this has a significant impact on the application of piezoelectric, air-coupled and EMAT ultrasound in NDT.

Generally, TPC requires some hardware resources and digital signal processing (DSP) to perform matched filter. However, in the case of SPC, some DSP expenditure can be replaced by the geometric structure. Some ultrasonic transducers, such as piezoelectric interdigital transducers and the meander line coil EMAT, have a geometric structure that forms a spatial matched filter [29,30]. Matthaei and Tancrell investigated the signal conversion process of a piezoelectric interdigital transducer using the spatial filter concept. They discovered that this structure can turn a long spatial wave packet into a narrow δ-like signal without any temporal signal processing [31,32]. Further, SPC has been used for interdigital transducer and surface acoustic wave (SAW) application [33,34]. In the case of the EMAT, line focusing EMAT (LF-EMAT) was presented to focus the shear vertical (SV) waves on a line inside the material, whose coil is a meander line coil with UES [35]. Similar to this structure, an SH-guided EMAT with a coil spacing that varies linearly in the propagation direction was used to generate a chirp ultrasonic signal [36]. A spatial phase encoding technique using 11-bit Barker code has been applied to RW-EMAT. The results indicated that the time resolution was improved by up to 72% with the SNR increase of 0.72 dB [37]. Our early research found that linear wavelength modulation (LWM) and nonlinear wavelength modulation (NLWM) coils can be used to realize SPC [38]. These two wavelength modulation methods are proposed with reference to the frequency modulation methods from TPC, such as linear frequency modulation (LFM) and nonlinear frequency modulation (NLFM) [26,27]. The typical EMAT with an ES coil is not suitable for wavelength modulation applications because it is not a wideband transducer due to its geometry. However, the LWM and NLWM coils are capable of realizing it and they show the potential to improve the signal quality of the RW-EMAT. These findings provided the early evidence for the possibility of SPC, which proves a potential approach to simplify the EMAT detection system while improving the strength, the time resolution and the SNR of the ultrasonic signal.

This paper investigates the SPC phenomena and proposes a new design of the RW-EMAT. Firstly, the mechanisms of TPC and SPC are analyzed, and the similarities and combinations of these two ways are explored. Secondly, the LWM and NLWM methods are proposed to design a new RW-EMAT with UES. The performance of the new coils is compared by their autocorrelation function. Finally, a finite element (FE) simulation model and an experimental system for the RW-EMAT are established to analyze the details of SPC. The effect of different coil configuration parameters on pulse compression is studied and discussed.

## 2. Theory

### 2.1. RW-EMAT with UES

**Figure 1:**
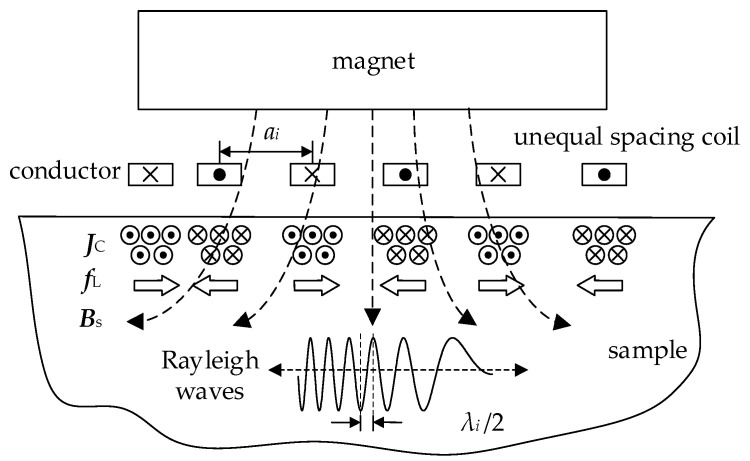
Mechanism of RW-EMAT with UES.

A typical RW-EMAT consists of a permanent magnet and a meander line coil with ES. In nonferromagnetic materials, only the Lorentz force mechanism is present. As shown in Figure 1, each conductor in the coil carries an altering current that has an opposite direction to the neighboring conductor. The Lorentz force ***f***_L_ is generated by the interaction of the static magnetic field ***B***_s_ and the eddy current ***J***_c_. The spacing distances between neighboring conductors have the same value, which is normally half the wavelength of the Rayleigh wave. Based on this structure, the phase difference of the Lorentz force under each conductor is π. Simultaneously, the wavelength of each wavelet in a same wave packet is identical. However, when a meander line coil with UES is used, the carrier wavelength becomes unequal and it varies with the coil spacing. The relationship between the coil spacing and wavelength can be expressed by Equation (1):

(1) $$a_{i}=\lambda_{i}/2,$$

where *i* represents the number of the conductor, *λ_i_* is the wavelength of the *i*-th half wave, and *a_i_* is the coil spacing distance between the *i*-th and the (*i* + 1)-th conductors, respectively.

### 2.2. Temporal Pulse Compression

**Figure 2:**
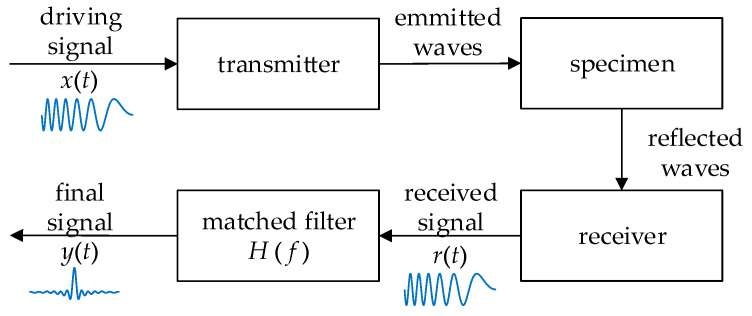
Schematic illustration of TPC process.

The process of the TPC for ultrasound NDT is shown in Figure 2. A driving signal *x*(*t*) is sent to the transmitter, and a received signal *r*(*t*) is obtained after wave propagation and reflection. Then, the final signal *y*(*t*) is obtained after *r*(*t*) passes through a filter whose transfer function is *H*(*f*). Generally, the echo signal in *r*(*t*) is similar to *x*(*t*); they have the same spectrum, which is denoted as *X*(*f*). Thus, *y*(*t*) can be described by Equation (2). For the TPC process, *x*(*t*) is a modulated signal while the filter is a matched filter whose transfer function satisfies as Equation (3):

(2) $$y(t)=\int_{-\infty }^{+\infty }X(f)H(f)e^{j2\pi ft}df\;,$$

(3) $$H(f)=kX^{*}(f)e^{-j2\pi ft_{0}}\;,$$

where *k* is a constant real number, *X*(*f*) is the spectrum of *x*(*t*), *t*_0_ is the time of flight (TOF) between the signal’s transmitting and receiving, and the superscript * represents the complex conjugate.

Then, *y*(*t*) obtains the maximum SNR, as described by Equation (4). In addition, the performance of the driving signal *x*(*t*) can be analyzed by the autocorrelation function Φ(*t*), as produced by Equation (5). The waveform of *x*(*t*) should be carefully designed so that the shape of its autocorrelation function is as similar to the delta function as possible.

(4) $$y(t)=k\int_{-\infty }^{+\infty }{\left|X(f)\right|}^{2}e^{j2\pi f(t-t_{0})}df\;,$$

(5) $$\Phi (t)=\int_{-\infty }^{+\infty }x\left(\tau \right)x\left(\tau +t\right)d\tau \;,$$

### 2.3. Spatial Pulse Compression

**Figure 3:**
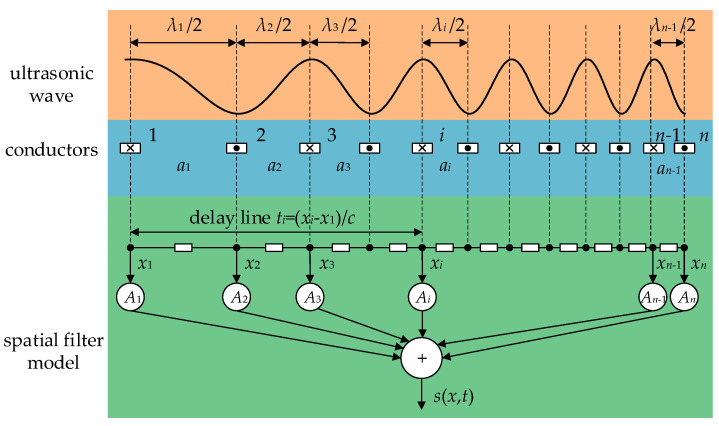
Spatial filter model for RW-EMAT with UES.

The effect of the RW-EMAT on the ultrasound signal is equivalent to a spatial filter. As shown in Figure 3, there are *n* conductors, labeled as 1, 2, …, *n*, with coordinates *x*_1_, *x*_2_, …, *x_n_*, respectively. The distance between the *i*-th and the (*i* + 1)-th conductor is *a_i_*. When an excitation current *f*(*t*) is applied to the coil, an ultrasonic wave source *A_i_f*(*t*) is generated under the *i*-th conductor. The spacing between two conductor forms into a delay line that causes the time delay of *t_i_* = *a_i_*/*c*. The response is calculated by Equation (6):

(6) $$s(x,t)=\sum_{i=1}^{n}A_{i}f(t-(x_{i}-x_{1})/c),$$

where *A_i_* depends on the energy conversion efficiency. In order to analyze the influence of coil distribution and simplify the process, it is assumed that *A_i_* = ±1 and the sign denotes the current direction.

**Figure 4:**
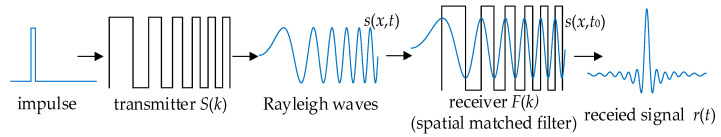
Schematic illustration of SPC process.

Figure 4 shows the schematic illustration of the SPC process using an RW-EMAT with a UES coil. Here, the Lorentz force distribution under the coil is assumed to be *s*(*x*), and its wavenumber domain function is calculated using spatial Fourier transform, as shown in Equation (7). Being excited by an impulse, the transducer launches a wave packet that is stretched to a series of wavelets. Assuming that the wave propagation time is *t*, the waveform distribution becomes *s*(*x*, *t*) = *s*(*x* − *ct*). Therefore, this distribution becomes *s*(*x*, *t*_0_) and *t*_0_ represents the time for the wave packet traveling below the receiver. The receiver is equivalent to a spatial filter, and the SPC occurs when the wavenumber domain function *F*(*k*) is the complex conjugate of *S*(*k*). Analogous to the TPC, a δ-like received signal *r*(*t*) is obtained and its performance can be analyzed using an autocorrelation function.

(7) $$S(k)=\int_{-\infty }^{+\infty }s(x)e^{-ikx}dx\;,$$

## 3. Method and Design

### 3.1. Wavelength Modulated Method

For ultrasound, the wavelength *λ* and wavenumber *k* satisfy *λ* = 2π/*k*, and the wave distribution is similar to the wavelength distribution. In order to realize the SPC process shown in Figure 4, two wavelength modulation methods are proposed with reference to TPC, such as LWM and NLWM, which are respectively based on LFM and NLFM.

Firstly, an LFM signal and an NLFM signal should be produced. LFM signal can be calculated by Equation (8). As for NLFM signal, it is generally no direct analytical expression and is numerically calculated based on a window function with a preset spectrum. For example, Equation (9) is a Hamming window:

(8) $$S(t)=\sin(2\pi f_{0}t+\frac{\pi \;B}{T}t^{2})\;,$$

(9) $$W(f)=\frac{T}{B}f+\frac{0.426T}{\pi }\sin(\frac{2\pi \;f}{B})\;,$$

where *S*(*t*) is the excitation signal for LFM, *W*(*f*) is the window function for NLFM, *f*_0_ is the initial frequency, *B* is the frequency bandwidth, and *T* is the time width.

Then, two new parameters, wavelength bandwidth *L* and wavenumber bandwidth *K*, are produced by Equations (10) and (11):

(10) L=cT ,

(11) K=B/c ,

where *c* is the velocity of Rayleigh wave. The conversion functions of wavelength modulation for LWM and NLWM are found as Equations (12) and (13), respectively:

(12) $$S(x)=\sin(k_{0}x+\frac{\pi \;K}{L}x^{2})\;,$$

(13) $$W(\lambda )=\frac{L}{cK\lambda }+\frac{0.426L}{\pi c}\sin(\frac{2\pi }{K\lambda })\;,$$

where *S*(*x*) is the ultrasonic wave distribution, *W*(*λ*) is the wavelength spectrum, *k*_0_ is the initial wavenumber.

**Figure 5:**
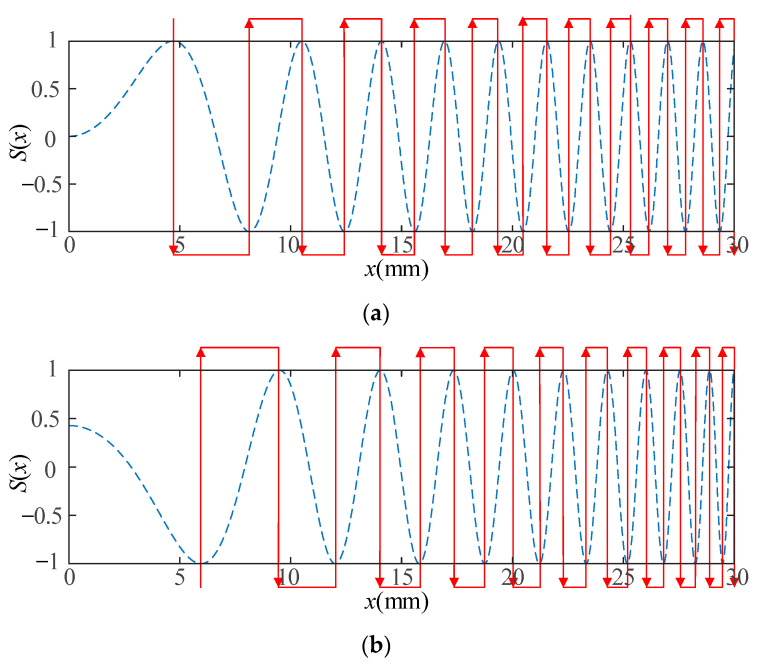
LWM and NLWM coil geometries for an aluminum sample: (**a**) LWM (*k*_0_ = 0 mm^−1^, *K* = 0.68 mm^−1^, *L* = 30 mm); (**b**) NLWM (*K* = 2 mm^−1^, *L* = 30 mm).

Finally, LWM and NLWM coils are designed once the values of *L* and *K* have been determined. Considering the geometric specifications, *L* influences the entire length of the coil, while *K* influences other features such as the number of conductors *N*, the main lobe width (MLW). Figure 5 shows the geometries of an LWM coil and an NLWM coil. The conductor coordinates correspond to the points whose value is ±1. Here, the red line represents the conductors, and the arrow represents the current direction. To simplify the calculation, we take *k*_0_ = 0. Because the calculation process performed by the LMW and NLWM methods are quite different, it is not meaningful to compare the values of *K* in the two methods.

### 3.2. Wavelength Modulation Parameters

The parameters *L* and *K*, mentioned in Section 3.1, determine the coil distribution *S*(*x*). A corresponding temporal function *S*(*t*) is obtained after replacing *x* to *ct* in *S*(*x*). Then, the autocorrelation function Φ(*t*) for *S*(*t*) can be used to evaluate the coil performance.

**Figure 6:**
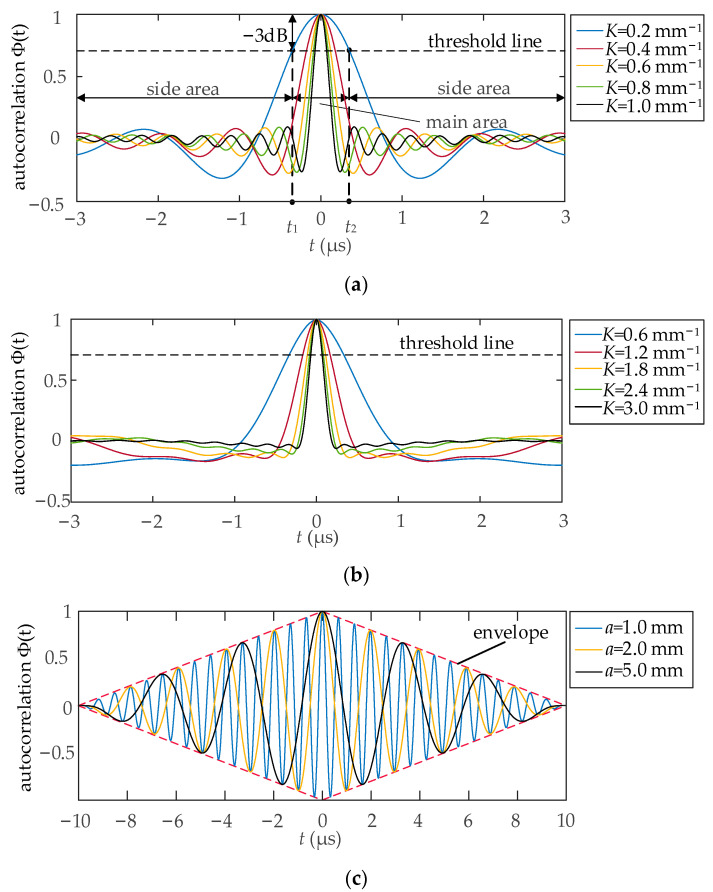
Autocorrelation functions Φ(*t*) with different structures and parameters: (**a**) LWM coils; (**b**) NLWM coils; (**c**) ES coils.

The entire coil width is often in the range of tens of millimeters. In this study, *L* is supposed to be between 20 to 60 mm. Simultaneously, different values of *K* are selected to compare the performances. For example, set *L* = 60 mm and the autocorrelation functions are presented in Figure 6a,b for LWM and NLWM coils, respectively. As a reference, the autocorrelation functions of ES coil are shown in Figure 6c. Figure 6a,b shows that when *K* increases, the main lobe becomes narrower and the side lobes become smaller. The red dashed line in Figure 6c shows the envelope of the autocorrelation function for the ES coil; it is approximately a triangular wave instead of a δ-like function. It means that this type of coil cannot be used for SPC although it forms a spatial filter.

In order to compare the performance of the autocorrelation function, main lobe width (MLW) and SNR are defined. The autocorrelation function with the blue curve in Figure 6a is used to illustrate the calculation of MLW and SNR. MLW is the time width between the two points (*t*_1_ and *t*_2_) at which the signal drops by 3 dB than its maximum value. The area within the user-defined range but outside of *t*_1_ and *t*_2_ is defined as the side area. Then, MLW and SNR are calculated by Equations (14) and (15), respectively [39]:

(14) $$\mathrm{MLW}=t_{2}-t_{1}\;,$$

(15) $${\mathrm{SNR}=10\log}_{10}\left[\left(\frac{1}{l_{M}}\int_{M}{\left|\Phi (t)\right|}^{2}dt\right)/\left(\frac{1}{l_{S}}\int_{S}{\left|\Phi (t)\right|}^{2}dt\right)\right],$$

where M represents the main area, S represents the side area, *l*_M_ represents the length of main area, *l*_S_ represents the length of side area, Φ(*t*) represents the autocorrelation function. Equation (15) is modified using common SNR calculation formulas. During the detection process, the main lobe signal is strong and the noise signal has little effect on it. However, the sidelobe signal and the noise signal will mix, and both are regarded as noise signals. Generally, smaller MLW and larger SNR are optimal, because it will make the waveform shape to be the δ-like function.

**Figure 7:**
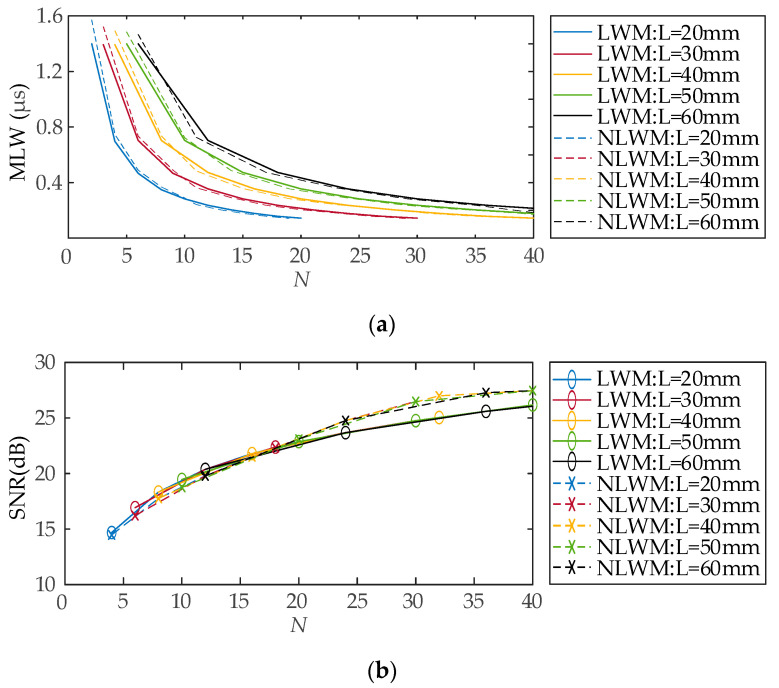
The effect of the conductor number on pulse compression performance: (**a**) MLW; (**b**) SNR.

Once *L* and *K* are selected, the LWM or NLWM coil structure is determined. In fact, changing *K* alters the number of conductors *N*. The effect of *N* on MLW and SNR is significant evidence to evaluate the performance. *N* can be calculated by counting the number of the points whose distribution function value is ±1. The results are presented in Figure 7. When *L* is the same, the MLW curves of the LWM and NLWM coils are very close as a function of *N*. As *N* increases, the MLW curves exhibit a declining tendency. The tendency decreases rapidly at first and then becomes flat when *N* becomes larger. The curves progressively move to the right as *L* rises. The compression performances of LWM and NLWM coils on the main lobe are quite similar, as the performance of SPC enhances as the conductor number increases when the length of RW-EMAT is determined. Figure 7b shows that the SNR for LWM and NLWM coils gradually rises as *N* increases, and the SNR curves are similar as *L* varies. The SNR of NLWM coils is larger than that of LWM coils when *N* > 20.

## 4. FE Simulation

### 4.1. Finite Element Model for RW-EMAT with UES

**Figure 8:**
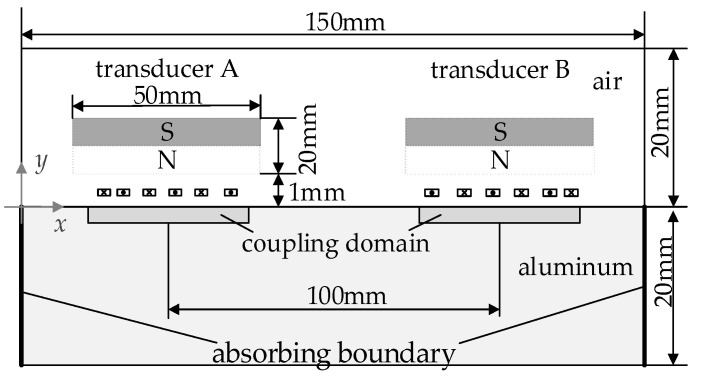
Geometry of the 2D FE model for RW-EMAT.

To investigate the SPC process, a two-dimensional FE model of RW-EMAT based on the Lorentz force mechanism was established with FE simulation software, COMSOL Multiphysics. Figure 8 shows the geometric structure and some geometric parameters of the model. Two transducers, A and B, are placed on an aluminum plate. Their coil can be of various kinds, including ES coil, linear coil, LWM coil or NLWM coil. The cross-sectional dimension of each conductor is 0.3 × 0.07 mm, and the lift-off distance is 0.5 mm.

In addition, this model was established using common modeling methods [40,41]. The permanent magnet material was NdFeB, and all the material characteristics in this model were taken from COMSOL. The magnetic field’s interface and the solid mechanics interface were added into the component node. The former interface was applied to all regions, while the latter interface was only applied to the specimen. The Lorentz coupling as a multi-physics branch was added to the coupling domain with a thickness of 5 times the skin depth. To reduce the boundary reflection, an absorbing boundary was placed on the left and right sides of the specimen. The mapped and triangular meshes were used in the model, whose minimum element size is 1 μm. The stationary study was set to solve the magnetic field of the magnet, and the time-dependent study was set to solve the dynamic magnetic field and solid mechanics field from 0 to 80 μs. As for the excitation source, a current pulse was supplied to the transmitting transducer, and its waveform could be selected from a variety of options, including narrow rectangular pulse, LFM or NLFM signal.

For an example, *L* = 30 mm is first chosen to calculate the Rayleigh waves. According to the results of the analysis in Section 3.2, selecting a design with a larger *N* is preferred. In fact, conductors need to maintain a certain width to provide mechanical strength; thus, they should not become too dense. As shown in Figure 7a, the contribution of increasing *N* to MLW improvement becomes less effective when *N* ≥ 20. As a result, the design with *N* = 20 is a good choice for the simulation. Then, two coils are produced: LWM coil (*k*_0_ = 0 mm^−1^, *K* = 0.68 mm^−1^, *L* = 30 mm) and NLWM coil (*K* = 2 mm^−1^, *L* = 30 mm).

### 4.2. Simulation Results

After two coils are produced, the simulation is carried out in two scenarios:

- Case 1: Coil A and B are the same structure (LWM or NLWM), and a rectangular current pulse with a peak value of 10A and a width of 0.5 μs is applied to transmitter A;
- Case 2: Coil A is a linear coil, while coil B has an LWM or NLWM structure. An LFM or NLFM current pulse corresponding to coil B’s structure is supplied to transmitter A.

**Figure 9:**
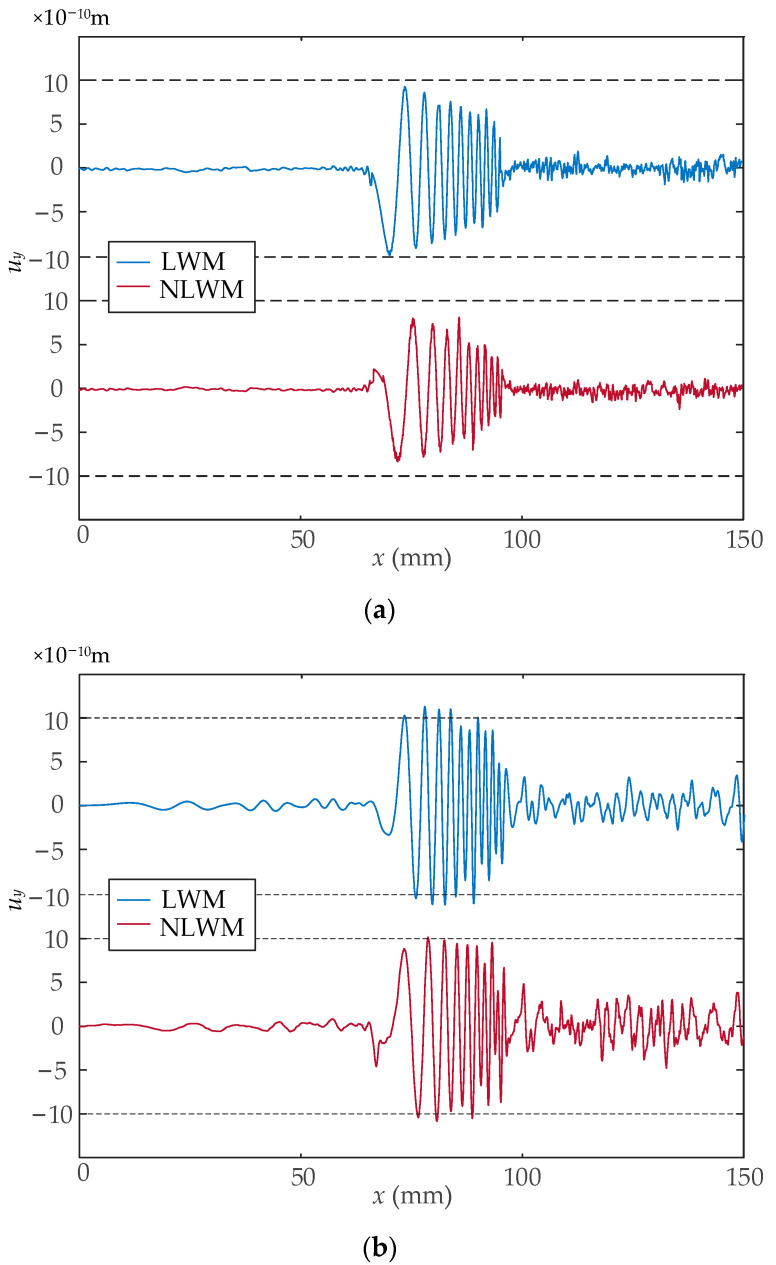
The *y*-direction displacement component along the surface of the sample: (**a**) case 1; (**b**) case 2.

Figure 9 shows the *y*-direction displacement component along the surface of the sample when *t* = 20 μs for case 1 and 2, respectively. The wave packets of Rayleigh waves retain a stable wavelength distribution during propagation. Figure 9a indicates that by using the LWM and NLWM coils, a rectangular pulse signal with width of 0.5 μs is stretched to a wave packet with a length of 30 mm. A similar wave package can be achieved by a linear coil with an LWM or NLWM current pulse. In addition, the distributions of Lorentz force and the acoustic field are quite similar with the coil geometry [38].

**Figure 10:**
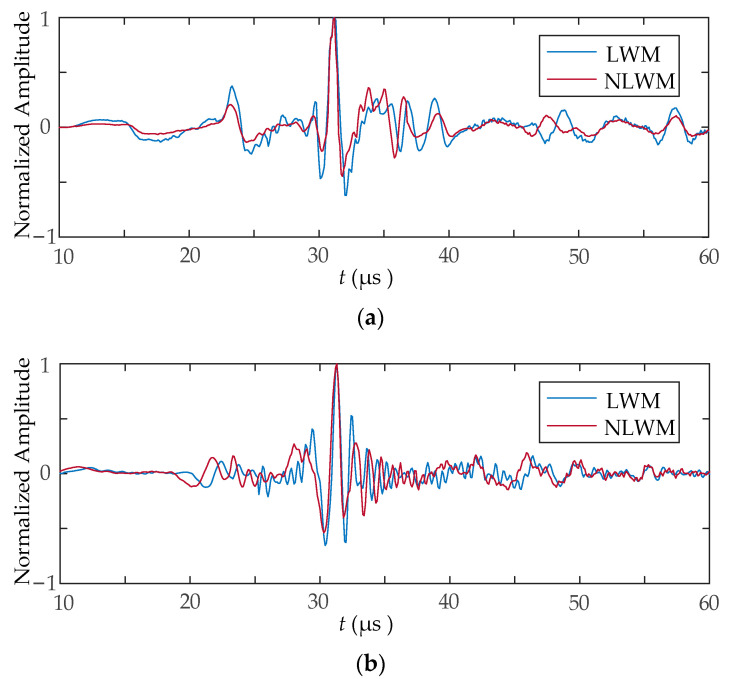
The current received signal: (**a**) case 1; (**b**) case 2.

Figure 10 shows the current signal received in coil B for case 1 and 2, respectively. Combining with the Rayleigh wave displacement in Figure 9, it can be seen that the wave packet with a length of 30 mm is compressed into a narrow pulse after the spatial matched filter. There are some sidelobes in the vicinity of the narrow main lobe. The shape of the sidelobes is affected by the waveform of the excitation signal and the geometry of the coil. The results in Figure 9 and Figure 10 verified the feasibility and validity of SPC.

## 5. Experimental Validation

**Figure 11:**
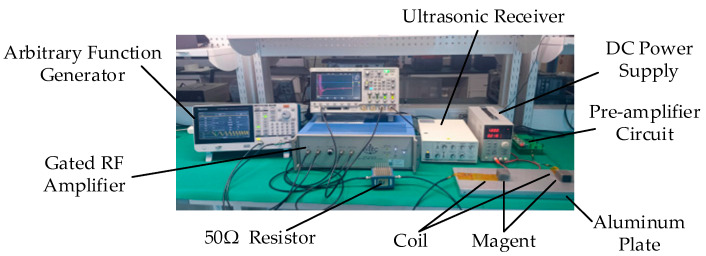
Picture of the experimental system for SPC test.

An RW-EMAT experimental system was established to evaluate the performances of SPC. The experimental setup is shown in Figure 11. It is built up based on some commercial instruments and a self-developed circuit. The arbitrary function generator (AFG31051, Tektronix, Beaverton, OR, USA) produced a rectangular pulse, LWM or NLWM signal to the gated RF amplifier (GA-2500A, Ritec, Pembrokeshire, UK). An excitation signal was obtained from the RF amplifier, which was applied to the transmitting transducer after passing through a 50 Ώ resistor load. Then, the received signal was connected to the ultrasonic receiver (DPR300, JSR Ultrasonics, New York, NY, USA), after passing through the self-developed pre-amplifier circuit, which was powered by a DC power supply. Then, an oscilloscope (DSO-X 3024A, Agilent, Santa Clara, CA, USA) and a current measurement system (TCPA300, Tektronix, Beaverton, OR, USA) were used to detect the experimental waveforms.

**Figure 12:**
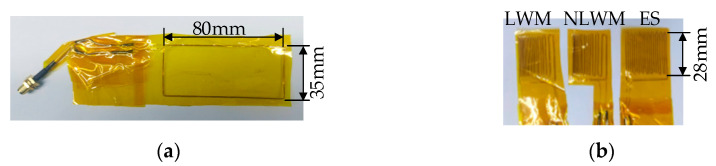
Picture of the coils: (**a**) linear coil; (**b**) LWM, NLWM and ES coil.

As shown in Figure 12, four different coils were hand-wound using copper enameled wires with 0.1 mm diameter. They were ES coil, linear coil, LWM coil and NLWM coil. The structure of the LWM or NLWM coil was the same as that in the simulation. The ES coil with 20 turns was prepared to compare its performance with the new design. The magnet for transducers was N52, with size of 35 × 40 × 25 mm. The aluminum specimen was 6061, with the size of 500 × 160 × 28 mm. The distance between the two transducer centers was 20 cm.

**Figure 13:**
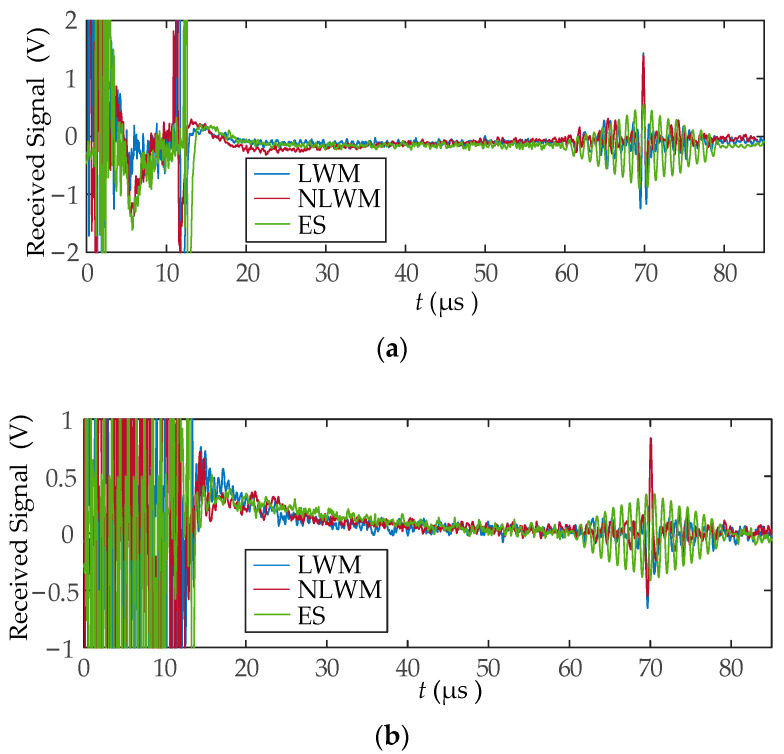
The received signals after the ultrasonic receiver: (**a**) case 1; (**b**) case 2.

**Figure 14:**
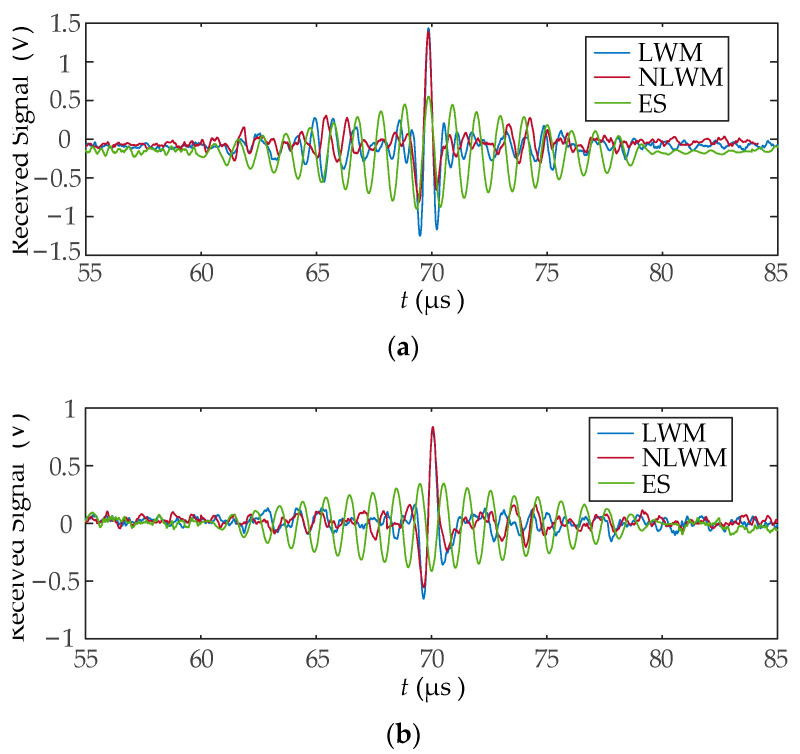
The received signals after enlargement: (**a**) case 1; (**b**) case 2.

Experiments were carried out by following the two cases that were defined in Section 4.2. In case 1, the excitation signal was configured as a rectangular wave with a peak current of 50A and a width of 0.5 μs. In case 2, an LFM or NLFM excitation pulse with a peak-to-peak current of 50A was supplied to the linear coil EMAT. The final received signals were averaged 8 times after being acquired by an oscilloscope, as shown in Figure 13. The enlarged received signals are shown in Figure 14. It shows that the amplitude of the received signals significantly increased after employing an LWM or NLWM coil. Simultaneously, the signal width was compressed to a spike-like pulse of less than 0.25 μs after SPC, which will be beneficial for the TOF measurement. The received signals in the range of 60~80 μs were used to calculate MLW and SNR, as shown in Table 1. Meanwhile, the signal amplitude ratio of LWM and NLWM coils to ES coil was calculated, as shown in Table 2. The received signal amplitude after LWM and NLWM coil was increased by 2.3~2.6 times. The SNR of received signal after LWM and NLWM coil was increased by 7.1~10.1 dB. These indicate that the LWM and NLWM coils have effectively achieved SPC, resulting in a received signal with a narrower width, higher amplitude and higher SNR than the ES coil. They have similar improvements, but the NLWM coil has a slightly higher SNR.

## 6. Conclusions

This paper proposes a new RW-EMAT based on the SPC. It converts the coil distribution from ES to UES and employs a matching excitation signal. This study explains the causes of the SPC using the spatial filter concept. The LWM and NLWM methods were proposed for designing the UES coil. Then, the characteristic parameters, MLW and SNR, were analyzed via the autocorrelation function. The results showed that the performance of these methods increases as the conductor number increases when the coil length is determined. The design results of LWM and NLWM with 20 conductors are presented. An FE model and an experimental system were established to study the performance of these new designs. The study proved the feasibility of the SPC and the effectiveness of the proposed methods. Experimental results confirmed that the signal amplitude is increased by 2.3~2.6 times by using the LWM or NLWM coil compared with the ES coil, and the 20 μs width signal can be compressed into a δ-like pulse of less than 0.25 μs. The SNR by using the LWM or NLWM coil is increased by 7.1–10.1 dB compared with the ES coil. These coils have similar improvements, but the NLWM coil has a slightly higher SNR. The proposed RW-EMAT can effectively enhance the strength, the time resolution and the SNR of the received signal. This is a significant benefit for EMAT application for the surface defect detection in the field of NDT.

It should be noted that the two examples of LWM and NLWM coils provided in this paper are for verification purpose, not to achieve the optimal outcome from the new design. If some other parameters of the RW-EMAT, such as the width of the magnetic field, the ratio of the thickness to the coil length and the conductor diameter, are carefully designed, the coil is expected to perform better.

The findings in this paper should be of interest to researchers in the area of surface defect detection of nondestructive testing, especially with the application of the Rayleigh wave EMAT. As the next step, SPC will be combined with TPC to generate a narrow δ-like acoustic field in the specimen, which is a potential application to control the excitation or reception mode of the guided waves in thin plates.

## Figures and Tables

**Table 1:** MLW and SNR in the experiment.

Case	MLW	SNR
LWM for case 1	0.18 μs	14.8 dB
NLWM for case 1	0.20 μs	17.3 dB
LWM for case 2	0.21 μs	16.8 dB
NLWM for case 2	0.22 μs	17.6 dB
ES for case 1	-	7.7 dB
ES for case 2	-	7.5 dB

**Table 2:** Signal amplitude ratio of the maximum value.

Case	LWM/ES	NLWM/ES
case 1	2.59	2.52
case 2	2.33	2.42

## Data Availability

Data are available upon request by contacting the corresponding author.
